# Adenosine A_2A_ Receptors in Striatal Glutamatergic Terminals and GABAergic Neurons Oppositely Modulate Psychostimulant Action and DARPP-32 Phosphorylation

**DOI:** 10.1371/journal.pone.0080902

**Published:** 2013-11-28

**Authors:** Hai-Ying Shen, Paula M. Canas, Patricia Garcia-Sanz, Jing-Quan Lan, Detlev Boison, Rosario Moratalla, Rodrigo A. Cunha, Jiang-Fan Chen

**Affiliations:** 1 Molecular Neuropharmacology Lab, Department of Neurology, Boston University School of Medicine, Boston, Massachusetts, United States of America; 2 Center for Neuroscience and Cell Biology, University of Coimbra, Coimbra, Portugal; 3 Instituto Cajal, Consejo Superior de Investigaciones Científicas, and Centros de Investigación Biomédica en Red, Instituto de Salud Carlos III, Madrid, Spain; 4 Robert Stone Dow Neurobiology Laboratories, Legacy Research Institute, Portland, Oregon, United States of America; 5 Faculty of Medicine, University of Coimbra, Coimbra, Portugal; INSERM/CNRS, France

## Abstract

Adenosine A_2A_ receptors (A_2A_R) are located postsynaptically in striatopallidal GABAergic neurons, antagonizing dopamine D_2_ receptor functions, and are also located presynaptically at corticostriatal terminals, facilitating glutamate release. To address the hypothesis that these two A_2A_R populations differently control the action of psychostimulants, we characterized A_2A_R modulation of cocaine-induced effects at the level of DARPP-32 phosphorylation at Thr-34 and Thr-75, c-Fos expression, and psychomotor activity using two lines of cell-type selective A_2A_R knockout (KO) mice with selective A_2A_R deletion in GABAergic neurons (striatum-A_2A_R-KO mice), or with A_2A_R deletion in both striatal GABAergic neurons and projecting cortical glutamatergic neurons (forebrain-A_2A_R-KO mice). We demonstrated that striatum-A_2A_R KO mice lacked A_2A_Rs exclusively in striatal GABAergic terminals whereas forebrain-A_2A_R KO mice lacked A_2A_Rs in both striatal GABAergic and glutamatergic terminals leading to a blunted A_2A_R-mediated facilitation of synaptosomal glutamate release. The inactivation of A_2A_Rs in GABAergic neurons reduced striatal DARPP-32 phosphorylation at Thr-34 and increased its phosphorylation at Thr-75. Conversely, the additional deletion of corticostriatal glutamatergic A_2A_Rs produced opposite effects on DARPP-32 phosphorylation at Thr-34 and Thr-75. This distinct modulation of DARPP-32 phosphorylation was associated with opposite responses to cocaine-induced striatal c-Fos expression and psychomotor activity in striatum-A_2A_R KO (enhanced) and forebrain-A_2A_R KO mice (reduced). Thus, A_2A_Rs in glutamatergic corticostriatal terminals and in GABAergic striatal neurons modulate the action of psychostimulants and DARPP-32 phosphorylation in opposite ways. We conclude that A_2A_Rs in glutamatergic terminals prominently control the action of psychostimulants and define a novel mechanism by which A_2A_Rs fine-tune striatal activity by integrating GABAergic, dopaminergic and glutamatergic signaling.

## Introduction

Striatal circuits, composed mainly of GABAergic medium spiny neurons (MSN), are the principal entry point of the basal ganglia and the primary site for processing of motor, motivational and cognitive behaviors [1]. MSN are driven by cortico-thalamic excitatory glutamatergic projections and modulated by nigral dopaminergic inputs. MSN project either directly (striatonigral MSN) or indirectly (striatopallidal MSN) to output nuclei [2]. Adenosine A_2A_ receptors (A_2A_R) are highly expressed in striatopallidal MSN where they antagonize dopamine D_2_ receptor (D_2_R) function [3]. In addition, A_2A_R are also located in striatal glutamatergic terminals [4] where they are involved in the modulation of glutamate release and corticostriatal synaptic transmission [5], [6], [7], [8]. Notably, blockade of A_2A_R in extra-striatal forebrain neurons attenuates behavioral responses to psychostimulants such as cocaine [9], amphetamine [10], [11] or L-DOPA [12]. This led us to propose that presynaptic A_2A_R in corticostriatal glutamatergic terminals exert their excitatory effects by facilitating glutamate release to counteract the inhibitory effect of postsynaptic A_2A_R in GABAergic MNS [3], [9]. This working model places A_2A_R in a unique position, integrating GABAergic, glutamatergic and dopaminergic neurotransmission to fine-tune striatal activity.

Dopamine- and cAMP-regulated phosphoprotein (DARPP-32) is a key signaling molecule coordinating MSN responsiveness, where its activity is regulated by its phosphorylation status on different residues, namely Thr-34 and Thr-75 [13]. The phosphorylation of striatal DARPP-32 at Thr-34 and Thr-75 is under tight control of dopamine, adenosine and glutamatergic signalling [13]. DARPP-32 phosphorylation at Thr-34 is controlled by the G_s_/G_i_-cAMP-PKA signalling pathway via D_1_ receptors (D_1_R) in the direct pathway and A_2A_R/D_2_R activation in the indirect pathway. DARPP-32 phosphorylation at Thr-75 in MSN is competitively inhibited by and inversely correlated with the activation of cAMP signalling and is additionally modulated by glutamate signalling via cdk5 kinase [14]. Studies with global [13] or striatal pathway-selective genetic deletion of DARPP-32 [15], [16], [17] confirmed that DARPP-32 activation in the direct and indirect pathways oppositely determines motor responses to psychoactive drugs. Specifically, the selective deletion of DARPP32 in the indirect pathway enhances psychomotor activity while the selective deletion of DARPP-32 in the direct pathway attenuates the psychomotor effect [15], [16]. Thus, Thr-34 and Thr-75 phosphorylation of DARPP-32 integrates the glutamatergic drive with dopaminergic extrinsic modulation as well as with intrinsic striatal modulation such as through adenosine [13]. We therefore hypothesize that A_2A_R in GABAergic and glutamatergic neurons modulates the action of psychostimulants through a putative opposite control of striatal DARPP-32 phosphorylation.

To test this hypothesis, we developed and characterized two cell type-selective A_2A_R knockout (KO) lines with selective deletion of A_2A_R either in inhibitory GABAergic striatopallidal neurons (striatum-A_2A_R KO, st-A_2A_R KO) or in excitatory glutamatergic cortical neurons in addition to GABAergic MSN (forebrain-A_2A_R KO, fb-A_2A_R KO). Their use allowed us to demonstrate that A_2A_Rs in GABAergic MSN and in corticostriatal glutamatergic terminals control the action of psychostimulants in opposite manners at the levels of (i) DARPP-32 phosphorylation; (ii) cocaine-induced c-Fos expression; and (iii) cocaine-induced psychomotor activity. This suggests that A_2A_R control the action of psychostimulants through the regulation of DARPP-32 phosphorylation (at Thr-34 and Thr-75) in striatopallidal neurons. Furthermore, these results define a novel function of A_2A_R in glutamatergic terminals and GABAergic striatopallidal neurons to fine-tune striatal neuronal activity and the action of psychostimulants through the integration of GABAergic, glutamatergic and dopaminergic signaling pathways.

## Results

### 1. Selective preservation of A_2A_R in glutamatergic but not GABAergic terminals in striatum- (but not forebrain-) A_2A_R KO mice

**Figure 1:**
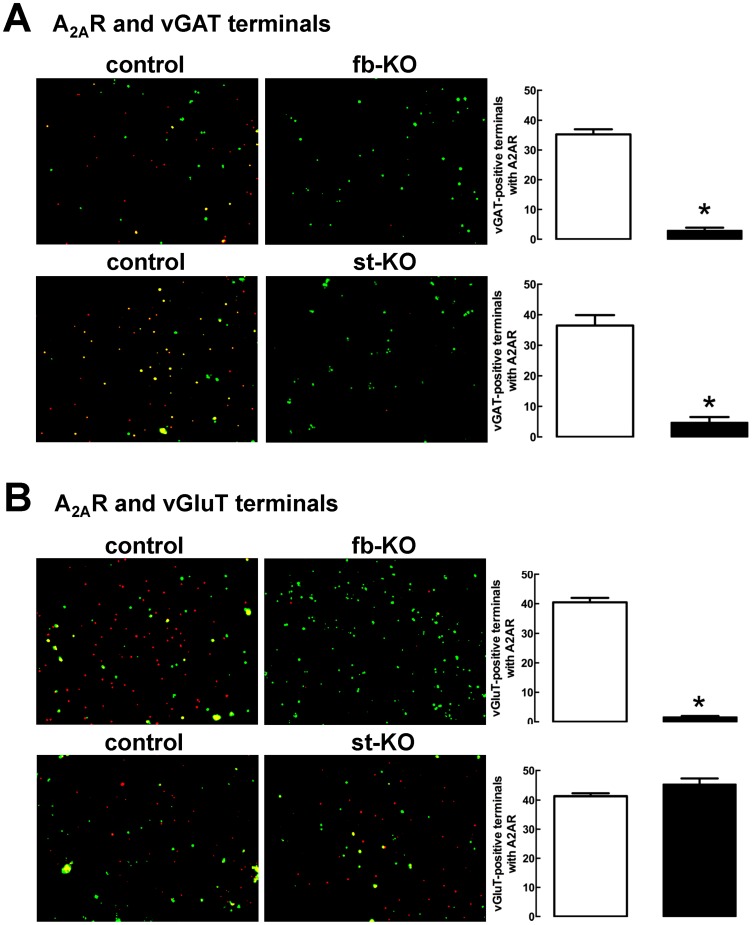
Deletion of A_2A_R immunoreactivity in glutamatergic terminals of forebrain-A_2A_R KO and GABAergic terminals of both forebrain A_2A_R - and striatum-A_2A_R KO mice. Detection and quantification of the percentage of GABAergic terminals (**A**, vGAT-positive) and glutamatergic terminals (**B**, vGluT1-positive) and from forebrain-selective-A_2A_R KO (fb-KO) or striatum-selective-A_2A_R KO (st-KO) mice and their wild type (WT) littermates (control) that are endowed with A_2A_R immunoreactivity. The bar graphs represent the percentage of vGluT1- or vGAT-immunopositive terminals that are also endowed with A_2A_R immunoreactivity (mean ± SEM, 3 fields per mouse, n = 4-6 animal per group). * p<0.05 *vs* corresponding WT littermates, using an unpaired Student’s *t* test. On the left side of each bar graph are shown representative immunocytochemistry photographs displaying the superimposed immunoreactivities of vGluT1 or vGAT (green) and of A_2A_R (red).

To demonstrate the selectivity of A_2A_R deletion in st-A_2A_R KO and fb-A_2A_R KO mice, we quantified A_2A_R immunoreactivity in glutamatergic (vesicular glutamate transporters type 1, vGluT1-positive) and GABAergic (vesicular GABA transporters, vGAT-positive) terminals from the striatum of st-A_2A_R KO, fb-A_2A_R KO and global A_2A_R knockout (gb-A_2A_R KO) mice as well as their corresponding wild-type (WT) littermates. Quantitative analysis revealed that A_2A_R immunoreactivity was depleted in GABAergic terminals from st-A_2A_R KO and fb-A_2A_R KO mice to background levels (n = 4–6 animals per group, p<0.05, unpaired Student’s *t* test) (Figure 1A) similar to these found in gb-A_2A_R KO mice (not shown). In contrast, A_2A_R immunoreactivity in glutamatergic terminals (about 50% of vGlut1-positive terminals contain A_2A_R, see [7]) was completely abolished in fb-A_2A_R KO mice and gb-A_2A_R KO mice (n = 4–6 animals per group, p<0.05, unpaired Student’s *t* test), but was selectively preserved in st-A_2A_R KO mice (n = 6, p>0.05, unpaired Student’s *t* test) due to the presence of presynaptic A_2A_R on corticostriatal terminals of extra-striatal glutamatergic neurons (Figure 1B). The preservation of presynaptic glutamatergic A_2A_R in st-A_2A_R KO mice was also consistent with the normal level of A_2A_R binding density in total membranes [9] and synaptosomal membranes (data not shown) of the cerebral cortex of st-A_2A_R KO mice. Together, these data demonstrate that A_2A_R in glutamatergic terminals of the striatum were selectively preserved in st-A_2A_R KO mice but abolished in fb-A_2A_R KO mice.

### 2. Selective preservation of the A_2A_R-mediated facilitation of glutamate release in synaptosomes from striatum-A_2A_R KO mice

**Figure 2:**
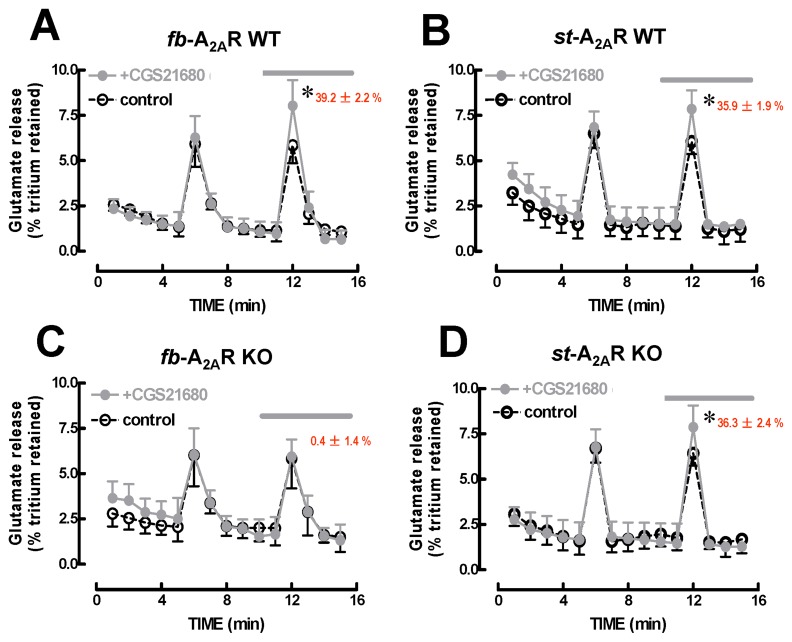
A_2A_R-mediated facilitation of ^3^H-glutamate release from striatal synaptosomes is abolished in forebrain-A_2A_R KO but preserved in striatum-A_2A_R KO mice. The A_2A_R selective agonist, CGS21680 (20 nM) facilitated the evoked ^3^H-glutamate release from striatal synaptosomes of fb-A_2A_R WT (n = 6, **A**), st-A_2A_R WT (n = 8, **B**) and st-A_2A_R KO (n = 4, **D**) but not fb-A_2A_R KO (n = 4, **C**). Each graph depicts the time course of tritium release enhanced by 20 mM K^+^ (evoked release, applied twice) in the absence (black symbols and lines) or presence of 20 nM CGS21680 (grey symbols and lines), as indicated by the horizontal lines. Indicates a significant (p<0.05, using a one-sample *t*-test against the hypothetical value of 0%) CGS21680-induced facilitation and the mean ± SEM facilitation is also indicated.

To examine the functional consequence of A_2A_R deletion in glutamatergic terminals in the striatum, we compared the ability of the selective A_2A_R agonist CGS21680 to facilitate glutamate release from striatal synaptosomes of fb-A_2A_R KO or st-A_2A_R KO mice. In the absence of CGS21680, the elevation of extracellular K^+^ concentration induced similar spike releases of glutamate from striatal synaptosomes from WT mice during two consecutive stimulation periods (ratio of 0.98 ± 0.02, n = 16). A supra-maximal but A_2A_R-selective concentration of CGS21680 (20 nM, present during the second period of stimulation) enhanced ^3^H-glutamate release by 38.2 ± 2.4% and 35.9 ± 1.9% from striatal synaptosomes of fb-WT and st-WT mice, respectively (n = 6-8, p<0.05 compared to 0%, one sample t-test) (Figure 2A and 2B). In contrast, CGS21680-mediated facilitation of ^3^H-glutamate release was completely abolished in striatal synaptosomes from fb-A_2A_R KO mice (n = 4, p>0.05 compared to 0%) (Figure 2C), but was unaffected in synaptosomes from st-A_2A_R KO mice (n = 4, p<0. 05 compared to 0%) (Figure 2D). These findings support the selective preservation of presynaptic A_2A_R function in glutamatergic terminals in st-A_2A_R KO but not fb-A_2A_R KO mice.

### 3. A_2A_R in glutamatergic terminals and GABAergic neurons oppositely modulate striatal DARPP-32 phosphorylation at Thr-34 and Thr-75

**Figure 3:**
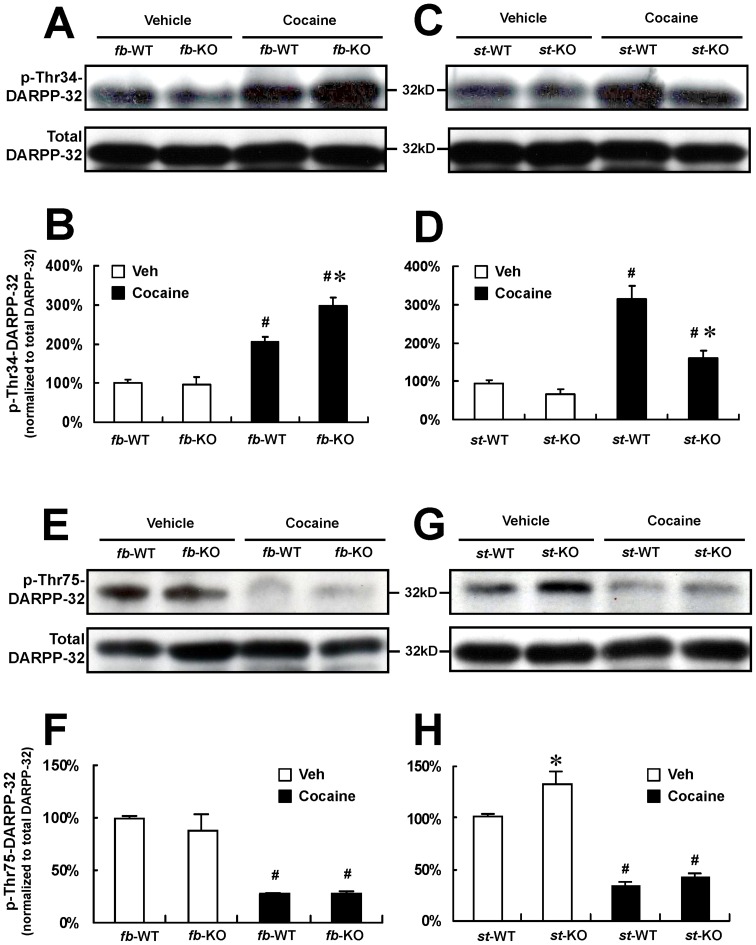
Cocaine-induced phosphorylation of striatal DARPP-32 at Thr-34 and Thr-75 are oppositely affected in striatum-A_2A_R KO and forebrain-A_2A_R KO mice. Western blot analysis of phosphorylated (p-Thr-34 and p-Thr-75) and total DARPP-32. Representative Western blots of striatal protein extracts from fb-A_2A_R KO and fb-WT (**A** and **E**), st-A_2A_R KO or st-WT mice (**C** and **F**). The levels of DARPP-32 phosphorylation (normalized with total DARPP-32 level) are shown as mean ± SEM and presented as percentage of the value for saline-treated WT mice, for p-Thr-34 DARPP-32 levels in *fb*-A_2A_R KO (n = 4, **B**) and *st-*A_2A_R KO (n = 6, **D**) and p-Thr-75 DARPP-32 levels in *fb*-A_2A_R KO (n = 4, **F**) and *st-*A_2A_R KO (n = 6, **H**). # p<0.05 comparing cocaine with saline treatment within same genotype, two-way ANOVA and a *post hoc* Bonferroni test; * p<0.05 comparing fb-A_2A_R KO or st-A_2A_R KO with their corresponding WT littermates with same treatment, two-way ANOVA *post hoc* Bonferroni test.

To determine the functional significance of the deletion of A_2A_R exclusively in GABAergic striatal neurons (in st-A_2A_R KO) and of its additional deletion in glutamatergic terminals (fb-A_2A_R KO), we evaluated the phosphorylation status of striatal DARPP-32 at Thr-34 in fb-A_2A_R KO mice (two-way ANOVA, drug effect: F_(1,12)_  = 95.765, p<0.001; genotype effect: F_(1,12)_  = 7.753, p = 0.017; drug x genotype: F_(1,12)_  = 9.034, p = 0.011) (Figure 3A, 3B) and in st-A_2A_R KO mice (two-way ANOVA, drug effect: F_(1,20)_  = 78.861, p<0.001; genotype effect: F_(1,20)_  = 25.924, p<0.001; drug x genotype: F_(1,20)_  = 12.508, p = 0.002) (Figure 3C, 3D). In addition, we also evaluated the phosphorylation status of striatal DARPP-32 at Thr-75 in fb-A_2A_R KO mice (two-way ANOVA, drug effect: F_(1,12)_  = 78.577, p<0.001; genotype effect: F_(1,12)_  = 0.600, p = 0.454; drug x genotype: F_(1,12)_  = 0.717, p = 0.414) (Figure 3E, 3F) as well as in st-A_2A_R KO mice (two-way ANOVA, drug effect: F_(1,12)_  = 624.116, p<0.001; genotype effect: F_(1,12)_  = 42.378, p<0.001; drug x genotype: F_(1,12)_  = 16.111, p = 0.002) (Figure 3G, 3H). Under basal condition (*i.e.* after treatment with vehicle), the level of DARPP-32 phosphorylation at Thr-75 or at Thr-34 was comparable between fb-A_2A_R KO mice and their fb-WT littermates (Figure 3B and 3F) (n = -4-6 per group, p>0.05, two-way ANOVA *post hoc* Bonferroni test). Interestingly, the deletion of A_2A_R in GABAergic striatopallidal neurons significantly increased the *basal* level of DARPP-32 phosphorylation at Thr-75 (Figure 3H) (n = 4 per group, p<0.05) together with a (mild) reduction of the basal level of DARPP-32 phosphorylation at Thr-34 (Figure 3D) in st-A_2A_R KO (but not fb-A_2A_R KO) mice (n = 6). These observations are consistent with a direct effect of postsynaptic A_2A_R in GABAergic neurons [18], [19]. Also in agreement with previous studies [18], [20], acute treatment with cocaine (25 mg/kg, i.p.) produced a marked increase of DARPP-32 phosphorylation at Thr-34 and a concomitant reduction of DARPP-32 phosphorylation at Thr-75 in WT mice (st-WT and fb-WT, Figure 3). As predicted from a direct, postsynaptic facilitatory effect of A_2A_R in GABAergic neurons, cocaine-induced DARPP-32 phosphorylation at Thr-34 was significantly attenuated in st-A_2A_R KO mice compared to their WT littermates (n = 6 per group, p<0.05 comparing cocaine with saline treatment) (Figure 3D). In contrast, the acute treatment with cocaine markedly increased DARPP-32 phosphorylation at Thr-34 in fb-A_2A_R KO mice compared to WT littermates (n = 4, p<0.05 comparing cocaine with saline treatment) (Figure 3A and 3B), consistent with a reduced glutamate release and dis-inhibition of glutamate suppression of DARPP-32 phosphorylation at Thr-34 in fb-A_2A_R KO mice [14]. Additional fluorescence immunohistochemistry using brain sections showed that DARPP32 phosphorylation at Thr-75 was markedly reduced 45 minutes after cocaine treatment in fb-WT and fb-A_2A_R KO mice (data not shown), a finding consistent with Western blot analysis. These findings demonstrated that, following cocaine treatment, presynaptic A_2A_R in glutamatergic terminals exert an opposite and predominant effect over postsynaptic A_2A_R in GABAergic neurons on striatal DARPP-32 phosphorylation at Thr-34 and Thr-75.

### 4. Cocaine-induced striatal *c-Fos* expression and psychomotor activity are enhanced in striatum-A_2A_R KO but attenuated in forebrain-A_2A_R KO mice

**Figure 4:**
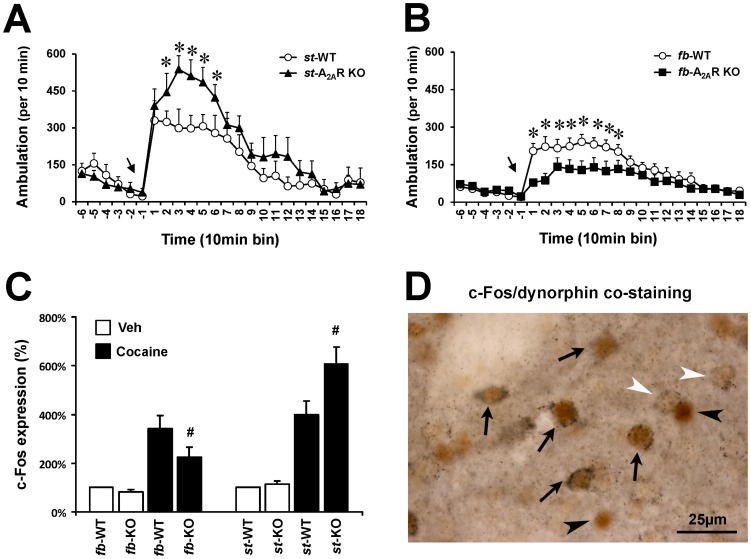
Cocaine-induced psychomotor activity and striatal c-Fos expression were attenuated in forebrain-A_2A_R KO but enhanced in striatal-A_2A_R KO mice. Ambulation was recorded for 180 min after injection of a single dose of cocaine (25 mg/kg, i.p.) or vehicle in fb-A_2A_R KO (n = 12, **A**) and in st-A_2A_R KO (n = 9, **B**) mice and their WT littermates (n = 8–12). The arrow indicates the time of injection and the data are mean ± SEM; *p<0.05, comparing fb-A_2A_R KO and st-A_2A_R KO groups to their corresponding WT group using two-way ANOVA and a *post hoc* Bonferroni test. (**C**) Cocaine-induced c-Fos expression in the striatum of fb-A_2A_R KO (n = 12) and st-A_2A_R KO (n = 9) and their corresponding WT littermates (n = 8–12) ^#^ p<0.05, comparing to corresponding wild-types with cocaine treatment, two-way ANOVA *post hoc* Bonferroni test. (**D**) Representative co-immunostaining of c-Fos with dynorphin. Black arrows indicate neurons co-stained with dynorphin and c-Fos; white arrow heads indicate neurons stained with dynorphin only (greyish brown) and black arrow heads indicate neurons stained with c-Fos (reddish brown). Scale bar  =  25 µm.

To evaluate the functional significance of the opposite modulation of striatal DARPP-32 phosphorylation by A_2A_R in GABAergic striatal neurons and in glutamatergic terminals, we compared cocaine-induced psychomotor activity and c-Fos expression, a measure of MSN activity, in the striatum of st-A_2A_R KO and fb-A_2A_R KO mice. Consistent with our previous reports [9], we found that cocaine (25 mg/kg, i.p.)-induced psychomotor activity was enhanced in st-A_2A_R KO (n = 9) but attenuated in fb-A_2A_R KO mice (n = 12) compared to their WT littermates (n = 8-12) (two-way ANOVA, drug effect: F_(1,24)_  = 91.892, p<0.001; genotype effect: F_(3,24)_  = 8.456, p<0.001; drug x genotype: F_(3,24)_  = 13.297, p<0.001) (Figure 4A and 4B). The opposite psychomotor effects of cocaine in st-A_2A_R KO and fb-A_2A_R KO mice were also paralleled by similar opposite effects of cocaine on c-Fos gene expression in the striatum of these two transgenic mouse strains. As expected, cocaine treatment (25 mg/kg, i.p.) increased c-Fos expression in the striatum of WT mice (st-WT and fb-WT, Figure 4C) to a similar extent. Interestingly, cocaine-induced striatal c-Fos expression was *enhanced* in st-A_2A_R KO mice (p<0.05, Student’s *t*-test, comparing with st-WT) (Figure 4C) but *reduced* in fb-A_2A_R KO mice compared to their corresponding WT littermates (p<0.05, Student’s *t*-test, comparing with fb-WT mice) (Figure 4C). Furthermore, double immunohistochemical analysis showed that the cocaine-induced increase of striatal c-Fos immunoreactivity in st-A_2A_R KO mice was restricted to dynorphin-positive cells (Figure 4D). As shown in Figure 4D, the majority of c-Fos-positive cells (black arrows) in the striatum were also stained with dynorphin, whereas some neurons were stained with dynorphin (white arrow heads) or c-Fos (black arrow heads) only.

**Figure 5:**
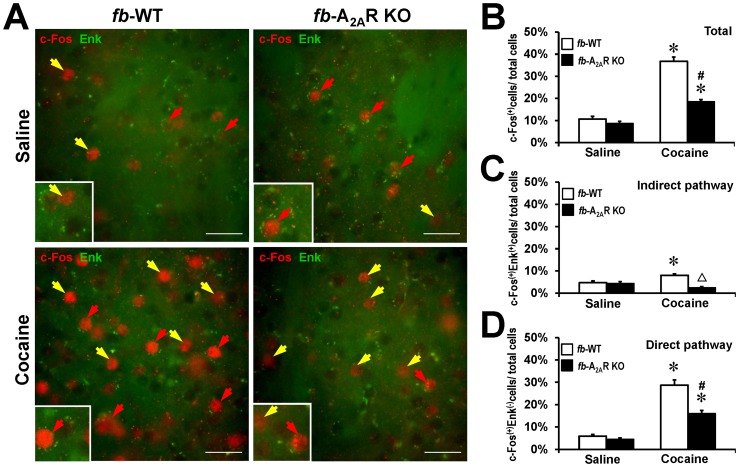
Immunofluorescence double staining of c-Fos and enkephalin in forebrain-WT and forebrain-A_2A_R KO mice after saline or cocaine treatment. (**A**) Representative merged images of immunofluorescence double staining of c-Fos (red) with enkephalin (Enk, green) in cocaine- *vs.* saline-treated fb-A_2A_R KO and fb-WT mice. (**B**) Quantitative analysis demonstrating the percentage of total c-Fos positive [c-Fos^(+)^]cells out of the total cells. (**C**) Quantitative analysis showing the percentage of c-Fos and Enk double positive [c-Fos^(+)^Enk^(+)^] stained cells out of the total cells. (**D**) Quantitative analysis demonstrating the percentage of c-Fos positive but Enk negative [c-Fos^(+)^Enk^(−)^] cells out of the total cells. Data in the bar graphs are mean ± SEM, n = 6-10 per group. * p< 0.05, *vs*. groups of same genotype with saline treatment; # p< 0.05 *vs*. cocaine-treated WT groups. ^△^ p< 0.05, *vs*. saline-treated fb-A_2A_R group. Scale bar  =  50 µm. Yellow arrows indicate neurons with c-Fos positive but Enk negative [c-Fos^(+)^Enk^(−)^] staining; Red arrows indicate neurons with c-Fos and Enk double positive [c-Fos^(+)^Enk^(+)^] staining.

Lastly, we performed double fluorescence immunohistochemistry to investigate if the cocaine-induced c-Fos expression mostly occurred in enkephalin (Enk)-positive or Enk-negative cells in fb-A_2A_R KO mice. The basal level of c-Fos expression in fb-A_2A_R KO mice was comparable with their WT littermates after saline injection (Figure 5, A and B), while enkephalin-positive cells constituted about 50% of the total cell population. Cocaine treatment markedly increased striatal c-Fos expression in fb-WT and fb-A_2A_R KO mice (two-way ANOVA, drug effect: F_(1,34)_  = 234.289, p<0.001; genotype effect: F_(1,34)_  = 70.643, p<0.001; drug x genotype: F_(1,34)_  = 56.521, p<0.001) (Figure 5, A and B). This induction largely occurs in Enk-negative cells (drug effect: F_(1,34)_  = 202.149, p<0.001; genotype effect: F_(1,34)_  =  33.480, p<0.001; drug x genotype: F_(1,34)_  =  21.888, p<0.001) (i.e. in the direct pathway, Figure 5D); this finding is consistent with our results using two color, sequential immunohistochemistry of c-Fos and dynorphin (Figure 4) and also agrees with previous reports that cocaine induces c-Fos expression predominantly in the D_1_R-containing striatonigral neurons (e.g. [17]). In fb-WT animals, we also observed a cocaine-induced c-Fos expression in the D_2_R-containing indirect pathway, likely attributed to a postsynaptic (striatopallidal) A_2A_R effect since cocaine-induced c-Fos expression was reduced in fb-A_2A_R KO mice (Figure 5C). Thus fb-A_2A_R KO mice displayed a reduced cocaine-induced c-Fos expression in the direct pathway as well as the indirect pathway, although the majority of cocaine-induced modifications of c-Fos expression in fb-A_2A_R KO mice were attributed to the direct pathway (see Figure 5). This finding suggests that the elimination of presynaptic glutamatergic A_2A_R mainly affects the direct pathway to control psychomotor activity and c-Fos expression.

## Discussion

### A_2A_R in glutamatergic corticostriatal terminals modulate psychomotor activity

Postsynaptic A_2A_R in GABAergic striatapallidal neurons are involved in the modulation of motor activity due to the concentrated expression of A_2A_R in striatopallidal neurons and their antagonistic interactions with D_2_R [21]. In addition, A_2A_R are also present and functional in presynaptic glutamatergic terminals that play a primordial role in driving striatal circuits [4], [5], [6], [8], [22]; however their role in the control of the action of psychostimulants remains largely unexplored, due to the low expression of the A_2A_R in the cerebral cortex and the inability to selectively manipulate A_2A_R in distinct cellular elements. Furthermore, it is currently unknown if the presynaptic A_2A_R might differentially affect the direct *versus* indirect pathways. The comparative analysis of the phenotypes of st-A_2A_R KO and fb-A_2A_R KO mice allowed us to dissect the effects of A_2A_R in glutamatergic terminals from those of A_2A_R in GABAergic striatopallidal neurons. Our main findings demonstrate that presynaptic A_2A_R in corticostriatal glutamatergic terminals facilitate glutamate release (by its presynaptic action alone or in combination with the action of postsynaptic A_2A_R) and play a predominant role in the control of DARPP-32 phosphorylation, striatal c-Fos expression, and consequent enhanced psychomotor activity upon cocaine exposure. Specifically, we showed that, in contrast to st-A_2A_R KO, fb-KO mice display a markedly reduced cocaine-induced c-Fos expression mainly in the MSN of the direct but also of the indirect pathway. Together with the finding that the deletion of A_2A_R in glutamatergic terminals in fb-A_2A_R KO abolished the A_2A_R-mediated enhancement of glutamate release, these results suggest that presynaptic A_2A_R control glutamate release, affecting the activity of both the direct and indirect pathways (with c-Fos expression as a marker for neuronal activity). This indicates that forebrain A_2A_R exert their control of cocaine action predominantly through the regulation of glutamate release, which challenges previous views attributing those actions to the control of the responsiveness of striatal GABAergic neurons. The most intriguing aspect of A_2A_R function in glutamatergic terminals is their ability to over-ride the effect of A_2A_R in striatopallidal neurons, which have a nearly 20-fold higher A_2A_R density [3]. This preferential engagement of A_2A_R in glutamatergic terminals is heralded by the observations that psychostimulants [23], [24], [25], [26] as well as NMDA receptor activation [27], [28] can enhance the local striatal extracellular levels of adenosine, preferentially near glutamatergic but not GABAergic terminals [25]. Thus, the pattern of generation of adenosine by psychostimulants may favor a preferential activation of presynaptic A_2A_R in corticostriatal terminals.

### A_2A_R in glutamatergic terminals and GABAergic neurons of the indirect pathway differentially modulate the action of psychostimulants through opposite control of DARPP-32 phosphorylation

Our current findings mechanistically dissociate the role of A_2A_R in glutamatergic terminals and in GABAergic neurons controlling DARPP-32 phosphorylation in the indirect pathway. Strikingly, the two different subsets of A_2A_R modulate the actions of psychostimulants via DARPP-32 phosphorylation in an opposite manner. In parallel with enhanced cocaine-induced c-Fos expression and psychomotor activity, the selective inactivation of A_2A_R in striatal GABAergic neurons (in st-A_2A_R KO mice) reduced DARPP-32 phosphorylation at Thr-34 and enhanced DARPP-32 phosphorylation at Thr-75. This modulation is consistent with a direct effect of A_2A_R on GABAergic striatopallidal neurons, since the inactivation of A_2A_R in GABAergic neurons reduces protein kinase A activity and in turn reduces DARPP-32 phosphorylation at Thr-34, with a parallel increase of DARPP-32 phosphorylation at Thr-75 [19], [29]. Furthermore, the increase of DARPP-32 phosphorylation at Thr-34 in fb-A_2A_R KO mice is best explained by the selective changes of DARPP-32 in the indirect pathway since the attenuation of cocaine-psychomotor activity is strongly correlated with enhanced DARPP-32 in the indirect pathway (not the direct pathway) as clearly demonstrated by the elegant work using cell-type specific DARPP-32 KO [15], [16]. Our findings are also in line with the concept that the striatopallidal pathway exerts a general inhibitory effect on behavior such as instrumental learning [30], psychostimulant activity [9], and aversive behavior [31], as revealed by selective destruction of the indirect pathway using targeted toxin expression [32] and by optogenetic silencing [33], [34].

However, the A_2A_R control of c-Fos expression in the striatum seems to result mainly from the c-Fos response in the direct pathway since we now demonstrated that cocaine-induced c-Fos expression was detected mainly in dynorphin-positive neurons. This effect could either result from recurrent collateral connections between striatopallidal and striatonigral MSN [35] or from an enhanced D_2_R-mediated release of endocannabinoids, which would decrease glutamate release from corticostriatal terminals projecting to both the indirect as well as the direct pathway [36]. This also explains the ability of A_2A_R to control D_1_R-mediated responses such as rotational behavior [37], [38], c-Fos expression in striatopallidal neurons [39] and DARPP-32 phosphorylation [29], [40]. In addition, the c-Fos expression may also be a secondary functional consequence of the enhanced psychomotor activity by selective deletion of A_2A_R in the indirect pathway. Although only derived from the use of a single dose of cocaine at single time point, the present findings nonetheless provide an important snapshot of the A_2A_R modulation of cocaine-induced molecular responses at the level of DARPP-32 phosphorylation and c-Fos expression in the striatum.

The additional elimination of A_2A_R from glutamatergic terminals in fb-A_2A_R KO mice reduced the basal Thr-75 phosphorylation of DARPP-32 and caused an enhancement of cocaine-induced Thr-34 phosphorylation of DARPP-32, in contrast to our findings in st-A_2A_R KO mice. This suggests that tonic activation of A_2A_R in glutamatergic corticostriatal terminals exerts opposite effects (compared to A_2A_R in GABAergic striatopallidal neurons) on striatal DARPP-32 phosphorylation. Since the major biochemical and neurochemical differences between fb-A_2A_RKO and st-A_2A_R KO mice is the deletion of A_2A_R in glutamatergic terminals (Figure 1) and the consequent abolishment of A_2A_R-facilitated glutamate release from striatal nerve terminals (Figure 2), the different regulation of DARPP-32 phosphorylation by A_2A_R in fb-A_2A_R KO mice likely results either from the impact of presynaptic A_2A_R on glutamate release alone or from the combined effect of presynaptic A_2A_R and postsynaptic A_2A_R actions, an issue that will require the use of selective deletions of A_2A_R in presynaptic glutamatergic corticostriatal terminals to be resolved. In fact, we are concluding that the differences between the phenotypes of fb-A_2A_R KO and st-A_2A_R KO mice are mostly due to the effects of presynaptic A_2A_R in glutamatergic corticostriatal terminals since the most evident differentiating factor in fb-A_2A_R KO mice is the deletion of presynaptic A_2A_R and the abolishment of A_2A_R-mediated facilitation of glutamate release. Since increased DARPP-32 phosphorylation at Thr-34 in the direct pathway is expected to produce enhanced cocaine psychomotor activity [15], [17], the increased DARPP-32 phosphorylation at Thr-34, together with the attenuation of cocaine-induced psychomotor activity in fb-A_2A_R KO mice strongly suggests that glutamate release by A_2A_R in corticostriatal terminals preferentially affects DARPP-32 phosphorylation in the indirect pathway. Conversely, fb-A_2A_R KO mice display an altered c-Fos expression in the direct and indirect pathways with the direct pathway being prominent one. Overall, the molecular and behavioral responses found in fb-A_2A_R KO mice suggest a selective modification of DARPP-32 phosphorylation in the indirect pathway and a prominent modification of cocaine-induced c-Fos expression in the direct pathway in tight correlation with cocaine-induced psychomotor activity. This is in line with the findings from cell-type specific deletion of DARPP-32, which showed that cocaine-induced psychomotor activity was attenuated by selective inactivation of DARPP-32 in the direct pathway [15]. While these results suggest that A_2A_R activity in glutamatergic terminals and GABAergic neurons may influence the action of psychostimulants by controlling DARPP-32 phosphorylation selectively in the indirect pathway, with the c-Fos response being secondary to the psychomotor effect, additional experiments are clearly warranted to clarify the cellular substrate linking the presynaptic A_2A_R control of glutamate release and its impact on psychomotor activity.

### Neurobiological and therapeutic implications

Based on the opposite phenotypes of cocaine-induced molecular and behavioral changes in st-A_2A_R KO and fb-A_2A_R KO mice, and their association with glutamatergic, GABAergic and dopaminergic systems at presynaptic and postsynaptic sites, we propose a new model for A_2A_R function in the control of striatal circuits: A_2A_R in glutamatergic terminals and GABAergic neurons provide a “fine-tuning” mechanism, whereby they integrate and regulate dopaminergic and glutamatergic signaling in the striatum. The integrated function of A_2A_R is accomplished through the opposing actions of A_2A_R in GABAergic striatal neurons (through A_2A_R-D_2_R antagonistic interactions) and in glutamatergic corticostriatal terminals (by modulating glutamate release). The novelty of this model is that the “fine-tuning” provided by A_2A_R may serve to prevent over- or under-stimulation of striatal neurons, and illustrates an essential aspect of the *integrated* function of the adenosine neuromodulation system [41]. Since decreased glutamatergic neurotransmission and increased dopaminergic activity contribute to the pathophysiology of schizophrenia and related psychiatric disorders, the ability of A_2A_R to integrate dopaminergic and glutamatergic systems indicates that adenosine acting at A_2A_R may modulate both positive (by preventing hyper-dopaminergic activity) and negative (by preventing hypo-glutamatergic activity) symptoms of schizophrenia [42]. Thus, the selective manipulation of presynaptic A_2A_R in glutamatergic terminals [43] may have a therapeutic value to manage a variety of neuropsychiatric behaviors such as anxiety, depression, psychosis and schizophrenia [44].

## Materials and Methods

### 1. Generation and genotyping of striatum-A_2A_R KO mice and forebrain-A_2A_R KO mice

Animals were handled according to the NIH Guide for the Care and Use of Laboratory Animals and in accordance with the protocol approved by the IACUC at the Boston University School of Medicine and by the Faculty of Medicine of the University of Coimbra. The Cre-loxP strategy was used to generate fb-A_2A_R KO and st-A_2A_R KO mice. The generation and genotyping of fb-A_2A_R KO mice has been described recently [10]. Briefly, transgenic mice expressing the Cre recombinase under control of the CaMKIIα gene promoter were crossbred with homozygous floxed (A_2A_R^flox+/+^) mice (F10 generation in congenic C57BL/6 background). Their Cre (+) A_2A_R ^flox+/+^ offspring display an A_2A_R deletion in postnatal forebrain neurons (including cortex, hippocampus and striatum). Similarly, homozygous floxed (A_2A_R^flox+/+^) mice (F5 generation in mixed 129-Steel and C57BL/6 background) were crossbred with Dlx5/6-Cre transgenic mice expressing Cre recombinase under control of the Dlx5/6 gene promoter, which is active exclusively in striatal neurons during development [45], [46], [47], to generate st-A_2A_R KO mice [Dlx5/6-Cre(+)A_2A_R^flox+/+^] mice [9]. Genotyping was conducted by 3 primer PCR analysis of tail DNA [10]. Fb-A_2A_R KO and st-A_2A_R KO mice were characterized for their selective *Adora2a* deletion in the forebrain (i.e., cortex, hippocampus, and striatum) [10], [48] or exclusively in striatal [9] neurons, as shown in our previous studies. The selectivity in these two lines was further validated by Cre-expression by X-gal staining of LacZ in a Rosa26 reporter transgenic line, PCR analysis of Cre-mediated *Adora2a* deletion, A_2A_R immunohistochemistry and ^3^H-ZM241385 radioligand binding of A_2A_R density [9], [10], [48], [49]. Our early studies showed that the behaviors of two WT genotypes [Cre(–)A_2A_R^flox+/+^ or Cre(+)A_2A_R^flox−/−^] were not distinguishable (data not shown) and so we used either WT type or in some cases two WT types were pooled in to one group referred to as simply st-WT or fb-WT, accordingly.

### 2. Drug treatments and psychomotor activity assessments

Before drug treatment, all mice were habituated in the testing environment and mice were injected with a single dose of cocaine (25 mg/kg, i.p.; Sigma, St. Louis, MO, USA). Horizontal locomotor activity was monitored for 180 min after drug administration and analyzed as described previously [9].

### 3. Glutamate release from striatal synaptosomes

^3^H-glutamate release experiments were performed as previously described after purification of striatal nerve terminals using a sucrose/Percoll fractionation method [22]. Briefly, nerve terminals were equilibrated at 37°C for 10 min, loaded with ^3^H-glutamate (0.2 µM, specific activity of 45 Ci/mmol, Amersham, Piscataway, NJ, USA) for 5 min at 37°C, washed, layered over Whatman GF/C filters and superfused with oxygenated Krebs solution for 20 min before starting collection of the superfusate. Synaptosomes were stimulated with 20 mM K^+^ at 3 min (S_1_) and 9 min (S_2_) after starting sample collection, triggering a release of tritium that was mostly ^3^H-glutamate, released in a Ca^2+^-dependent manner [22]. The A_2A_R agonist CGS21680 (Tocris, Bristol, UK), tested at a concentration that is supra-maximal but selective to activate A_2A_R [22], was added 2 min before S_2_ onwards and its effect was quantified by modification of the S_2_/S_1_ ratio compared to control chambers. Normalized facilitation by CGS21680 of the K^+^-evoked ^3^H-glutamate release was tested by the one-sample *t*-test against the hypothetical value of 0% compared to paired control experiments carried out in the same batch of nerve terminals in the absence of added drugs. P ≤ 0.05 was considered to represent a significant difference.

### 4. Immunocytochemical detection of A_2A_R in glutamatergic and GABAergic nerve terminals

Striatal nerve terminals were purified through a discontinuous Percoll gradient and platted over poly-L-lysine-coated cover-slips for immunocytochemical analysis, using antibodies that were previously validated [22], [50]. Permeabilized nerve terminals were incubated for 1 hour with rabbit anti-A_2A_R (1∶500, Upstate Biotechnology, Lake Placid, NY, USA), and guinea pig anti-vesicular GABA transporters (vGAT, 1∶1,000, Calbiochem, San Diego, CA, USA) or guinea pig anti-vesicular glutamate type 1 transporters (vGluT1, 1∶1000, Chemicon, Temecula, CA, USA) antibodies followed by a 1 hour incubation with different AlexaFluor-labeled secondary antibodies (1∶2,000, Molecular Probes, Leiden, The Netherlands), which did not yield any signal in the absence of the corresponding primary antibodies. After washing and mounting onto slides with Prolong Gold Antifading (Invitrogen, Eugene, OR, USA), preparations were visualized in a Zeiss fluorescence microscope and analyzed with MetaFluor 5.0. Each coverslip was analyzed by counting three different fields and in each field a total amount of 150 individualized elements excluding elements based on their insufficient or excessive pixel intensity and excessive size, as previously described [22], [50]. Note that this approach can only globally distinguish glutamatergic from GABAergic terminals, but the anti-vGluT1 and anti-vGAT antibodies used cannot distinguish between the different types of glutamatergic terminals (projecting to the direct or indirect pathways) or GABAergic terminals (direct projections or collaterals).

### 5. Western blot analysis of DARPP-32 phosphorylation at Thr-32 and Thr-75

DARPP-32 immunoreactivity was analyzed as previously described [51] with modifications. Mice were sacrificed by decapitation (45 min after i.p. injection of vehicle or drug) and their heads were immediately immersed in liquid nitrogen for 6 sec. The striata were rapidly (within 20 sec) dissected out on an ice-cold surface, sonicated in 750 µL of 2% sodium dodecylsulfate, and boiled for 10 min. After protein determination, 30 µg protein from each sample was loaded and separated by Western blot to quantify phospho-DARPP-32 (Thr34) (1∶1000, antibody kindly provided by Dr. Greengard) and phospho-DARPP-32 (Thr75) (1∶1,000, Cell Signaling, Danvers, MA, USA), normalized to total DARPP-32 immunoreactivity (1∶1,000, Cell Signaling).

### 6. Immunohistochemistry of c-Fos expression and double labeling of c-Fos with dynorphin or enkephalin

**Sequential antibody detection of c-Fos and dynorphin.** Free-floating brain coronal sections (30 µm) were double stained immunohistochemically with anti-c-Fos and anti-dynorphin polyclonal antibodies using standard avidin–biotin procedures following a sequential antibody detection protocol as described previously [52], [53]. For this procedure, the first antibody, *i.e.*, a goat anti-dynorphin polyclonal antibody (1:200, sc-46313, Santa Cruz, CA, USA) was detected first, using immunoperoxidase staining enhanced with 0.08% nickel ammonium sulfate, which yields a dark grayish color. After completion of the first staining, the same sections were incubated with an avidin/biotin blocking solution in order to block free avidin/biotine sites from the first biotinylated goat anti-rabbit IgG antibody. Then, sections were processed for immunolabeling with the second primary antibody, *i.e.*, a rabbit anti-c-Fos polyclonal antibody (1∶5,000, PC-38, Calbiochem) following standard protocols using DAB, yielding a bright brown color. This method has been repeatedly shown to lack cross-labeling [52], [53]. Moreover, the nuclear localization of c-Fos staining, as opposed to the cytoplasm/neuropil staining of dynorphin, makes it easy to differentiate the two types of staining.

**Fluorescence double immunohistochemistry of c-Fos and enkephalin.** Coronal brain sections (30 µm) were double stained overnight with primary antibodies, namely rabbit anti-c-Fos polyclonal antibody (1∶5,000, PC-38, Calbiochem) and mouse anti-enkephalin monoclonal antibody (1∶50, sc-47705, Santa Cruz). After washing, slices were incubated for 1 hour at room temperature in a solution containing a goat anti-rabbit secondary antibody, conjugated to Cy3 (1∶750, 111-165-144, Jackson Immuno Research, West Grove, PA, USA) and a goat anti-mouse secondary antibody, conjugated to FITC (1∶200, 115-095-166, Jackson Immuno Research). The sections were then washed 3 times and mounted on gelatin-coated slides and cover slipped with Vectashield fluorescent mounting medium with DAPI (H-1200, Vector Lab, Burlingame, CA, USA).

### 7. Statistical analysis

Statistical comparisons between st-A_2A_R KO vs st-WT or fb-A_2A_R KO vs fb-WT were analyzed (independently for their different genetic backgrounds) using a paired or unpaired Student’s *t* test, according to the experimental design. To determine the effect of genotype, drug treatment and their interaction, we applied a two-way ANOVA for repeated measurements followed by Bonferroni *post hoc* comparison.
